# Noncanonical PI3Kγ signaling addiction is a therapeutic target in a subset of leukemias

**DOI:** 10.1016/j.xinn.2024.100706

**Published:** 2024-09-30

**Authors:** Yu Zhang, Qian Kong, Yonglong Zhang

**Affiliations:** 1 Department of Clinical Laboratory, Shanghai Sixth People’s Hospital Affiliated with Shanghai Jiao Tong University School of Medicine, Shanghai 200233, China; 2 Department of General Surgery, Shanghai Jiao Tong University Affiliated Sixth People’s Hospital, Shanghai 200233, China

## Main text

Acute myeloid leukemia (AML) is a devastating disease with a median age of diagnosis of over 60 years. Thus, most AML patients are not eligible for intensive chemotherapy. Relapse remains common in patients with AML despite intensive therapy, which significantly compromises therapeutic options. Although several targeted therapies have been licensed for treating AML subsets bearing specific mutations and variant proteins essential for AML propagation, no actionable molecular targets are available for most AML patients. This has been hindered by disease heterogeneity and a general lack of effective biomarkers that could be used to predict cellular vulnerabilities to selected therapies for various AML subsets. A recent work published in *Nature* by Luo et al. identifies a phosphatidylinositol 3-kinase γ (PI3Kγ)-dependent therapeutic vulnerability in a subset of AML and provides a novel intervention strategy.

The PI3Ks are a family of heterodimeric lipid kinases consisting of a catalytic and an adaptor subunit. Mammals express three classes of PI3K enzymes (I, II, and III), and class I PI3Ks (PI3Kα, PI3Kβ, PI3Kδ, and PI3Kγ) convert phosphatidylinositol (4,5) bisphosphate to phosphatidylinositol (3,4,5) trisphosphate to activate signaling pathways implicated in cell proliferation, survival, and metabolism, whereas class II and class III PI3Ks are involved in membrane trafficking. The diverse functions of class I PI3Ks are controlled by four catalytic subunits (p110α, p110β, p110δ, and p110γ), which are encoded by PIK3CA, PIK3CB, PIK3CD, and PIK3CG, respectively. Class I PI3Ks are further classified into class IA (p110α, p110β, and p110δ) and class IB (p110γ) based on their distinct regulatory subunits. While class IA PI3Ks share a regulatory subunit of p85α/β, class IB p110γ binds to either a p101 or p87 subunit, encoded by PIK3R5 and PIK3R6, respectively.

PI3Kα and PI3Kβ are expressed ubiquitously, while expression of PI3Kδ and PI3Kγ is restricted in immune cells. PI3Kα and, to a lesser extent, PI3Kβ, are frequently mutated in several human solid cancers and have been the focus of extensive studies and drug development for decades. Several PI3Kα or PI3Kδ inhibitors have been approved by the US Food and Drug Administration, and certain mutant-selective PI3Kα inhibitors are being tested in clinical trials for treating PIK3CA-mutated cancer patients.^1^ By contrast, PI3Kγ is less studied in cancers, such as AML. Early studies showed that PI3Kγ in macrophages induces immune suppression of solid tumors and that targeting PI3Kγ potentiates checkpoint blockade therapy. However, how cancer cell-intrinsic PI3Kγ functions has been largely left unclarified, which compromises additional therapeutic options for targeting PI3Kγ in certain subtypes of AML without actionable mutations or defined targets, although several targeted therapies are approved for patients with specific, aberrantly activated pathways.

Recent work published in *Nature* by Luo et al. identifies PI3Kγ signaling addiction in a subset of leukemias that could be targeted by a PI3Kγ inhibitor.^2^ PIK3R5 and PIK3CG, which encode a p101 regulatory and p110 catalytic subunit of PI3Kγ complex, are essential for the survival of a leukemia subset including blastic plasmacytoid dendritic cell neoplasm (BPDCN) and AMLs with high expression of PIK3R5 via a genome-wide CRISPR interference screening. Further analysis suggests a dependency of PIK3R5 and PIK3CG exclusively in leukemia cells and confirms an expression pattern of PIK3R5 and PIK3CG specifically enriched in BPDCN and a subset of AML compared with most AML cell lines. Unlike AML cell lines with low PIK3R5 and PIK3CG, cells highly expressing PIK3R5 or PIK3CG are more sensitive to their loss and treatment with the PI3Kγ inhibitor eganelisib. This observation is further supported by the increased vulnerability to eganelisib treatment in patient-derived tumor xenografts (PDXs) highly expressing PIK3R5. Interestingly, the PIK3R5-high leukemia subset does not associate with specific mutational profiles but shows a molecular signature of innate immune response. Indeed, PIK3R5 is induced in PIK3R5-low leukemia cells by Toll-like receptor agonist and inflammatory cytokines, such as interferon α (IFNα) and IFNγ. Such a regulation renders leukemia PDXs sensitive to the PI3Kγ inhibitor eganelisib, indicating a leukemia subset selectively addicted to PI3Kγ signaling potentially driven by innate inflammatory signaling and elevated PIK3R5. The transcription factor SPI1 is then identified, which mediates PIK3R5 transactivation, which is considered to further stabilize P110γ to enhance PI3Kγ signaling. While sufficient evidence confirms the regulation of PIK3R5 by SPI1, it is still unclear whether the observed SPI1-PIK3R5 axis occurs in the leukemia subset defined by elevated PIK3R5. Additional assays are required to examine whether SPI1 is related to elevated PIK3R5 in this leukemia subset. Unlike p110α and its regulatory subunit of p85α/β, a reciprocal control between p110γ and p101 protein turnover is identified, highlighting the unique regulation and cell-specific expression of the PI3Kγ complex. Importantly, AML cells, upon PI3Kγ inhibition, are characterized by a defect in oxidative phosphorylation, which is typically required for AML cells and chemoresistant AML in particular, implying an alternative strategy targeting oxidative phosphorylation for treating AML. Conversely, nuclear factor κB signaling is potently activated in cells following PI3Kγ inhibition, which partially represses oxidative phosphorylation and mediates PI3Kγ dependency.

Luo et al. next sought to determine the major signaling output of leukemias downstream of the PI3Kγ complex by phosphoproteomics. Intriguingly, in AML and BPDCN cells, PI3Kγ inhibition by eganelisib or genetic loss inhibited the noncanonical substrate PAK1, but not AKT (also known as protein kinase B, PKB). PAK1 is known to be activated by RAC1, and indeed, PI3Kγ inhibition attenuates RAC1/PAK1 association. A known phosphorylation site at serine 144 (S144) of PAK1 by RAC1 is further confirmed, which mediates PAK1 function downstream of PI3Kγ signaling. In support of this observation, PAK1 (S144) phosphorylation correlates with PIK3R5 and PIK3CG expression in AML patients and their responses to eganelisib, indicating a critical event of PAK1 phosphorylation for establishing PI3Kγ addiction. It’s worth noting that PAK1 is not identified as a dependency gene from CRISPRi screening, implying other mechanisms potentially involved downstream of the PI3Kγ complex. Also, it is a challenging yet interesting topic how PI3Kγ signaling relies on PAK1 instead of the canonical PI3K substrate AKT. In contrast to the observation of two associated studies published recently in *Nature Cancer* and *Blood*, they showed that PI3Kγ signaling goes through AKT signaling to confer AML cell fitness and leukemia stem cell maintenance.^3^^,^^4^ Despite distinct cell lines used and context-dependent effects, it is crucial to understand this discrepancy, which may help improve therapeutic strategies and patient stratification. Nevertheless, the unconventional PI3K substrate PAK1 downstream of PI3Kγ signaling is an additional option of PAK1 inhibition for the aberrantly activated PI3Kγ subset of AML.

Considering that the leukemia subset addicted to PI3Kγ signaling observed here recapitulates the biological characteristics of cytarabine-resistant leukemia cells, Luo et al. continued to explore the translational potential of the combination of eganelisib with cytarabine in leukemia. Indeed, targeting PI3Kγ is even more effective than cytarabine in PIK3R5-elevated AML cells without clear side effects, and eganelisib synergizes with cytarabine to inhibit tumor growth *in vivo*. Using additional leukemia PDX models, Luo et al. surprisingly found that eganelisib not only considerably extends the survival time of mice bearing PIK3R5-high leukemia PDX but also resensitizes those with low PIK3R5 to cytarabine. This led to the discovery of four G protein-coupled purinergic receptors that are elevated in cytarabine-resistant PIK3R5-low leukemia cells and stimulate PI3Kγ-PAK1 signaling that contributes to eganelisib sensitivity. This important finding holds implications for treating cytarabine-resistant leukemia cells via targeting PI3Kγ or G protein-coupled purinergic receptors.

**Figure 1:**
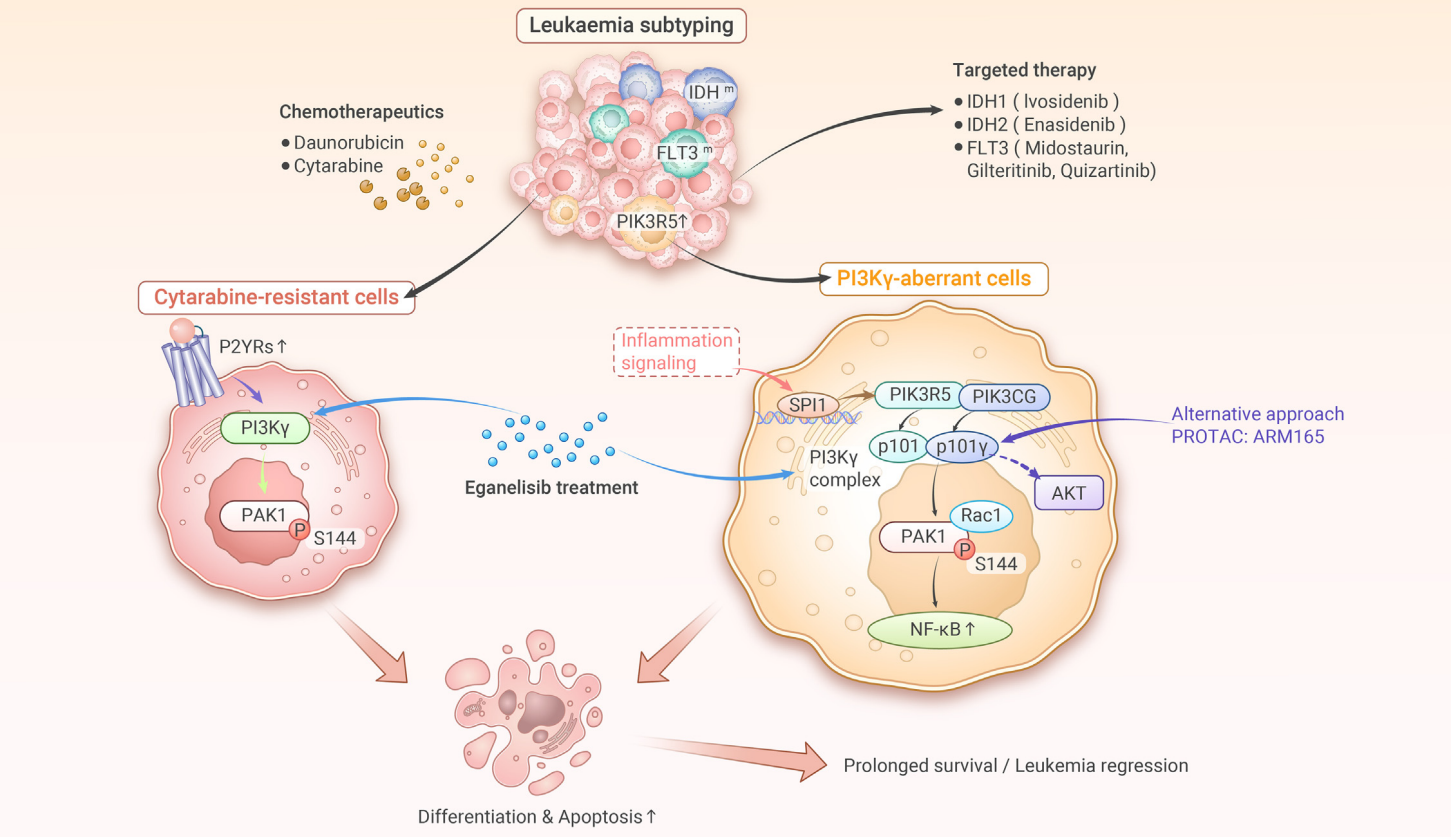
Targeting noncanonical PI3Kγ signaling for treating AML Despite significant advances in the landscape of genetic alterations in AML resulting in several targeted therapies, how the vulnerabilities of AML without mutations to specific therapies are dictated has remained largely unknown. This is further accentuated by the emergence of chemoresistance. The discovery that selective PI3Kγ complex dependency in a subset of AML signals through the unconventional PAK1 kinase provides a novel intervention via PI3Kγ inhibition using eganelisib for treating AML and overcoming chemoresistance.

The successful characterization of PI3Kγ-PAK1 signaling dependency provides the rationale for treating AML without actionable mutations or a defined mechanistic target through PI3Kγ inhibition (Figure 1).^3^^,^^4^ Given that eganelisib is being tested in several clinical trials for solid tumors, it is very interesting to assess its safety and therapeutic efficacy in patients with AML.^5^ Overall, this study provides a proof of concept for targeting gene dependency via PI3Kγ to treat the PIK3R5-restricted monocytic lineage of AML and provides novel strategies for synergizing conventional therapies to overcome chemoresistance.

## Acknowledgments

This work was supported by the 10.13039/501100001809National Natural Science Foundation of China (82472802 and 82073258) and Natural Science Foundation of Shanghai (23ZR1448600).

## Declaration of interests

The authors declare no competing interests.
